# Temperature-Induced ICD Malfunction in a Patient With Brugada Syndrome

**DOI:** 10.1016/j.jaccas.2025.105065

**Published:** 2025-09-17

**Authors:** Surachat Jaroonpipatkul, Apichai Pokawattana

**Affiliations:** Division of Cardiology, Rajavithi Hospital, College of Medicine Rangsit University, Bangkok, Thailand

**Keywords:** Brugada syndrome, hyperthermia, T-wave oversensing

## Abstract

**Background:**

Brugada syndrome is associated with life-threatening arrhythmias and requires implantable cardioverter-defibrillator (ICD) placement, but inappropriate shocks remain a clinical challenge.

**Case Summary:**

A 43-year-old male firefighter with Brugada syndrome and an automated ICD experienced both appropriate and inappropriate shocks. The shocks occurred primarily during duty in high-temperature environments. Device interrogation revealed the false shocks were due to T-wave oversensing triggered by elevated body temperature. True ventricular tachycardia episodes were also recorded during sleep. The ICD was reprogrammed, and temperature exposure precautions were advised.

**Discussion:**

This case demonstrates the impact of environmental heat exposure on electrocardiogram morphology and ICD performance in patients with Brugada syndrome. It emphasizes the need for individualized programming and occupational risk assessment.

**Take-Home Messages:**

T-wave oversensing from elevated temperature can trigger inappropriate ICD shocks in patients with Brugada syndrome. Individualized ICD settings and environmental risk management are essential in high-risk occupations.

## Graphical Abstract

**Figure:**
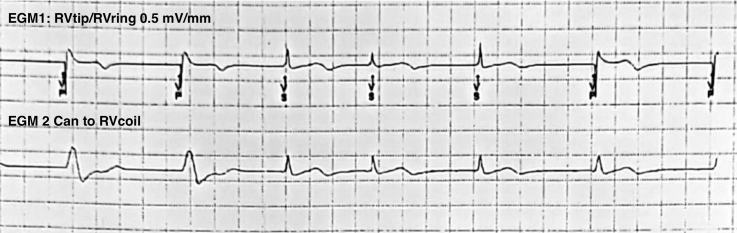

## History of Presentation

A 43-year-old Thai man with a known history of Brugada syndrome presented to the outpatient clinic after reporting multiple implantable cardioverter-defibrillator (ICD) shocks that occurred during his firefighting duties. He had remained asymptomatic during these episodes, without associated chest pain, palpitations, or syncope. Physical examination was unremarkable, with normal vital signs and no signs of heart failure or neurological deficits.

**Take-Home Messages**

• T-wave oversensing from elevated temperatures can trigger inappropriate ICD shocks in patients with Brugada syndrome.
• Individualized ICD settings and environmental risk management are essential in patients with high-risk occupations.

## Past Medical History

**Figure 1:**
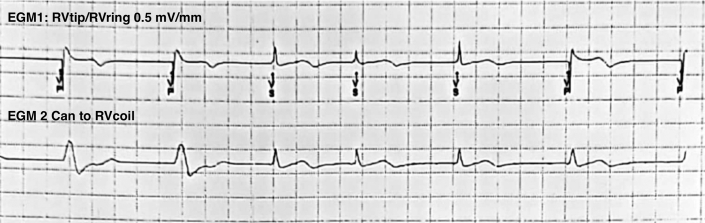
Baseline Intracardiac Electrogram The upper tracing represents the RV tip to RV ring (EGM1) channel, and the lower tracing represents the can to RV coil (EGM2) channel. The recording scale is 0.5 mV/mm. EGM = electrogram; RV = right ventricular.

The patient was diagnosed with Brugada syndrome in April 2015 after an episode of out-of-hospital cardiac arrest, during which he was found unresponsive and required successful defibrillation after ventricular fibrillation was documented. Subsequent work-up, including coronary angiography and brain imaging, was unremarkable. An ICD was implanted for secondary prevention. In 2017, he experienced bradycardia-related inappropriate shocks, prompting an adjustment of the base pacing rate from 40 to 50 beats/min. However, the multiple shocks that were experienced before led to the rapid depletion of the device battery. Consequently, in October 2018, the automated ICD generator was replaced. The new device comprised a coil lead (6947M Sprint Quattro dual; Medtronic) and a generator (Mirro MRI VR, Medtronic). The patient's baseline electrogram is shown in Figure 1.

## Differential Diagnosis

The differential diagnosis for the patient's ICD shocks included true ventricular tachyarrhythmias associated with Brugada syndrome, T-wave oversensing, lead dysfunction, electromagnetic interference, and myopotential oversensing. However, the absence of symptoms, the stable lead parameters, and the preserved sensing thresholds further supported the likelihood of inappropriate shocks due to oversensing rather than true arrhythmia or device malfunction.

## Investigations

**Figure 2:**
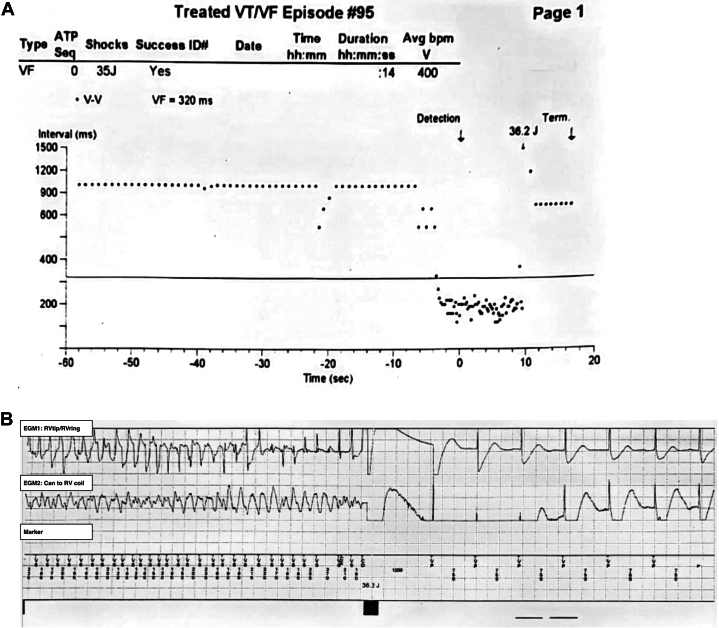
Examples of Episode With True Device Shock (A) Interval plot of ventricular sensed events showing a ventricular fibrillation (VF) detection with subsequent ICD shock. (B) Intracardiac EGMs during the same episode. The top channel represents the RV tip to RV ring, the middle channel is the can to RV coil, and the bottom panel shows the marker channel. The signal scale is 0.5 mV/mm. EGM = electrogram; ICD = implantable cardioverter-defibrillator; RV = right ventricular.

**Figure 3:**
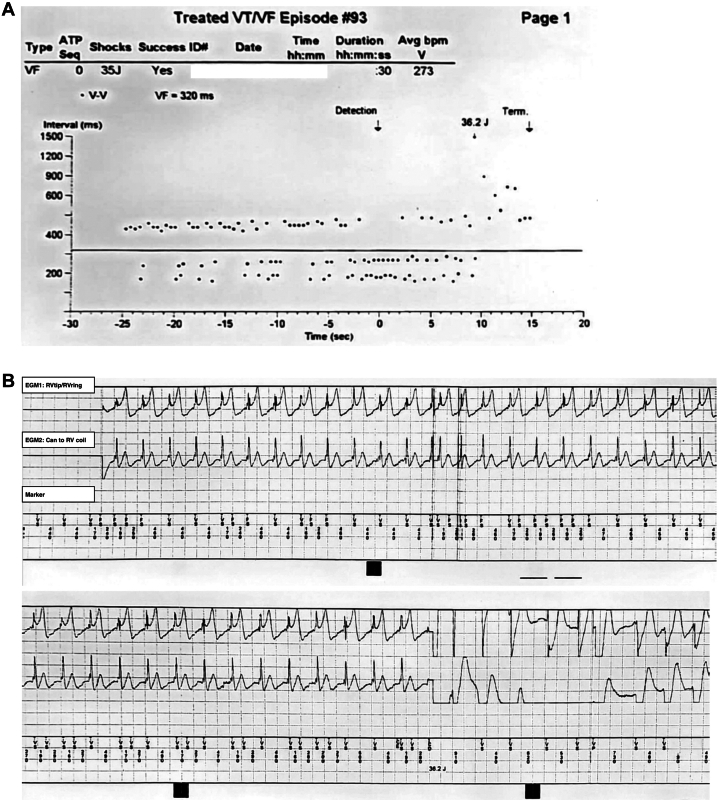
Episode of False Shock (A) Interval plot of sensed ventricular events interpreted as ventricular fibrillation (VF) by the ICD, with detection and shock delivery at 36.2 J. (B) Intracardiac EGMs during the same episode. The upper channel shows the RV tip to RV ring signal, the middle channel shows the can to RV coil signal, and the bottom panel is the marker channel. The scale is 0.5 mV/mm. Oversensing of large-amplitude T waves led to double counting and inappropriate shock therapy. Abbreviations as in Figure 2.

Device interrogation revealed a total of 10 ventricular fibrillation and ventricular tachycardia detections since February 2024, of which 9 resulted in ICD-delivered shocks and 1 spontaneously terminated. Electrograms demonstrated both true arrhythmic episodes during sleep and inappropriate detections during high-temperature exposure (Figures 2 and 3). During episodes of inappropriate therapy, the T-wave amplitude was notably elevated. The electrogram, recorded from the right ventricular (RV) tip to the RV ring channel using a scale of 0.5 mV/mm, showed baseline T-wave amplitudes of approximately 1.0 to 1.5 mV, which increased to approximately 3.75 mV during periods of high ambient temperature. This substantial rise in T-wave amplitude likely contributed to T-wave oversensing and double counting by the ICD, resulting in inappropriate shock delivery. Lead parameters, including pacing and defibrillation impedance, sensing thresholds, and battery status, were within normal limits, ruling out lead fracture or dysfunction. Baseline electrocardiogram (ECG) demonstrated a type 1 Brugada pattern. Serum potassium was not measured at the time of the shock events, however all routine follow-up laboratory results demonstrated stable and normal potassium levels, with no evidence of electrolyte abnormalities during the entire follow-up period.

## Management

The patient's ICD settings were reviewed and reprogrammed to reduce sensitivity to T-wave oversensing. Adjustments were made to the sensing threshold and detection parameters. Given the association between high ambient temperatures and inappropriate shocks, he was advised to avoid environments with excessive heat and to pause his firefighting duties. His medication regimen was modified, with low-dose quinidine (400 mg/d) to reduce arrhythmia risk related to Brugada syndrome. He received education on recognizing signs of true arrhythmias versus device-related events and was scheduled for follow-up with remote monitoring.

## Outcome and Follow-Up

After device reprogramming and lifestyle modifications, the patient reported no further inappropriate ICD shocks. True arrhythmic episodes also decreased in frequency. He remained asymptomatic and adhered to environmental precautions and medical therapy. Subsequent device interrogations confirmed stable lead parameters and appropriate sensing, without further oversensing episodes. The patient continues regular follow-up through in-person visits and remote monitoring.

## Discussion

Brugada syndrome is a genetic disorder characterized by distinctive ECG findings and an elevated risk of sudden cardiac death. Patients with Brugada syndrome frequently require ICD implantation to manage life-threatening arrhythmias.^1^ The case presented involves a 43-year-old Thai man with Brugada syndrome who experienced both appropriate and inappropriate ICD shocks, particularly during periods of increased body temperature associated with his work as a firefighter.

ECG changes in Brugada syndrome can be influenced by various factors, such as fever, and the morphology of the ECG can change from type 1 to type 2 or other patterns, such as bundle branch block.^2^ Temperature changes can cause ICD malfunctions, including electromagnetic interference and true arrhythmias, owing to various factors.^3^ An increase in ICD shocks can adversely affect patient outcomes. Several studies from Asian countries, including Japan, Korea, and Taiwan, have indicated that heat may affect ICD shocks and have shown that high temperatures are associated with increased sudden cardiac death and cardiovascular mortality.^4^

The mechanisms underlying the occurrence of sustained ventricular tachyarrhythmias (VTAs) during thermal stress are complex and multifactorial.^5^ During heat stress, increased skin blood flow and volume depletion due to sweating can lead to reduced coronary blood flow and increased blood viscosity. Additionally, heart rate and cardiac contractility tend to rise, exacerbating the imbalance between myocardial oxygen supply and demand. These factors can further strain the cardiovascular system. Arterial thrombosis and disturbances in the autonomic nervous system may also elevate the risk of VTAs.^6^ High ambient temperatures are linked with a greater risk of out-of-hospital cardiac arrest and cardiovascular mortality.^7^ Most sudden deaths in this context are attributed to VTAs, which can occur even in individuals without pre-existing cardiac conditions.^8^ Research from Korea and Japan indicates that VTA incidents peak during the summer months in patients with conditions such as right ventricular dysplasia, Brugada syndrome, and early repolarization syndrome.^4^

In this patient, changes in body temperature and corresponding ECG changes were documented in the ICD records. The relationship between body temperature and ECG changes, particularly T-wave amplitude, is critical to this case. The T-wave represents the repolarization phase of the ventricles and is sensitive to various physiological and pathological conditions. Changes in T-wave morphology can be influenced by factors such as electrolyte imbalances, myocardial ischemia, and alterations in autonomic tone.^9^

One significant factor influencing T-wave morphology is body temperature. Hyperthermia, or increased body temperature, can affect the ECG by altering the ion channels involved in the repolarization process.^10^ This can lead to changes in T-wave amplitude and morphology, which an ICD can misinterpret as ventricular tachycardia or ventricular fibrillation.

In this case, the patient's occupation as a firefighter exposed him to high temperatures, which likely contributed to the observed ECG changes and false ICD shocks. The elevated T-wave amplitude during periods of high body temperature led the ICD to double count the heart rate, mistaking benign T-wave changes for ventricular tachycardia or ventricular fibrillation. This intermittent T-wave oversensing was detected by the ICD because of high-temperature exposure. This case underscores the importance of considering environmental and occupational factors in managing patients with ICDs and Brugada syndrome. Furthermore, the case highlights the need for careful programming of ICD parameters to minimize the risk of inappropriate shocks. Adjusting sensitivity settings and incorporating temperature monitoring could potentially reduce the incidence of false shocks triggered by nonpathological ECG changes.

## Conclusions

This case illustrates the complex interplay between body temperature and ECG morphology in patients with Brugada syndrome and ICDs. Increased body temperature can lead to significant T-wave changes, potentially causing inappropriate ICD shocks. These findings emphasize the need for individualized ICD programming and consideration of environmental factors in managing such patients. With the increase in global temperatures from climate change, particularly in Southeast Asian countries, proper ICD settings and adjustments could become increasingly important. Future research should focus on developing strategies to differentiate between actual arrhythmic events and temperature-induced ECG changes to improve the safety and efficacy of ICD therapy in patients with Brugada syndrome.

## Funding Support and Author Disclosures

The authors have reported that they have no relationships relevant to the contents of this paper to disclose.
